# Recent Developments in Combination Immunotherapy with Other Therapies and Nanoparticle-Based Therapy for Triple-Negative Breast Cancer (TNBC)

**DOI:** 10.3390/cancers16112012

**Published:** 2024-05-25

**Authors:** Gantumur Battogtokh, Onyinyechi Obidiro, Emmanuel O. Akala

**Affiliations:** Center for Drug Research and Development, Department of Pharmaceutical Sciences, College of Pharmacy, Howard University, Washington, DC 20059, USA; gantumur.battogtokh@howard.edu (G.B.); onyinyechi.obidiro@bison.howard.edu (O.O.)

**Keywords:** TNBC, combinatorial therapy, nanotechnology, immunotherapy, chemotherapy

## Abstract

**Simple Summary:**

Among the different types of breast cancers, triple-negative breast cancer (TNBC) has a poor prognosis and is the most difficult type to treat by monotherapy due to the absence of common targeting receptors. In our review, we focused on the current advanced combination therapies, which include immunotherapy and chemotherapy, immunotherapy and gene therapy, and nanotechnology-based combination therapies for TNBC treatment. Additionally, we discussed the therapeutic outcomes of in vivo studies and clinical trials of the combination therapies.

**Abstract:**

Triple-negative breast cancer (TNBC), lacking specific receptors found in other breast cancer subtypes, poses significant treatment challenges due to limited therapeutic options. Therefore, it is necessary to develop novel treatment approaches for TNBC. In the last few decades, many attempts have been reported for alternative tools for TNBC treatment: immunotherapy, radiotherapy, targeted therapy, combination therapy, and nanotechnology-based therapy. Among them, combination therapy and nanotechnology-based therapy show the most promise for TNBC treatment. This review outlines recent advancements in these areas, highlighting the efficacy of combination therapy (immunotherapy paired with chemotherapy, targeted therapy, or radiotherapy) in both preclinical and clinical stages and nanotechnology-based therapies utilizing various nanoparticles loaded with anticancer agents, nucleic acids, immunotherapeutics, or CRISPRs in preclinical stages for TNBC treatment.

## 1. Introduction

Based on the Global Cancer Observatory reported in 2018, two million cases of breast cancers have been diagnosed worldwide [1,2]. Several subtypes of breast cancers, including estrogen receptor (ER)-positive, progesterone receptor (PR)-positive, human epidermal growth factor 2 receptor-positive (HER-2), and triple-negative breast cancer (TNBC) are known [3,4]. The TNBC is a heterogeneous group of tumors lacking ER, PR, and HER-2. TNBC is present in 10–20% of breast cancer cases, and it is most often connected to African-Americans of a younger age, with higher grade, mitotic index, and more advanced stages at diagnosis [5]. Due to the absence of major targetable receptors, chemotherapy is the only preferred approach for the systemic treatment of TNBC to enhance disease outcomes. The survival rate post-metastasis is shorter in comparison with other breast cancer subtypes. Due to these difficulties, the TNBC has limited treatment options and a poor response rate. TNBC can be classified into seven molecular subtypes, which include basal-like subtypes (BL1 and BL2), mesenchymal stem-like (MSL), unstable (UNS), immunomodulatory (IM), mesenchymal (M), and luminal androgen receptor (LAR) [6].

TNBC can be diagnosed early through various methods, including palpation, mammography, ultrasound, magnetic resonance imaging (MRI), and immunohistochemistry (IHC) [7]. Three different types of analysis are used to diagnose breast cancer in daily medical practice: (A) radiological/image examination (which encompasses mammography, magnetic resonance imaging (MRI), ultrasonography, and others); (B) clinical examination and (C) immunohistopathological examinations [4]. 

Depending on the type and stage of cancer, TNBC patients are treated with different therapies. These therapies include surgery, chemotherapy, radiotherapy, immunotherapy, stem cell therapy, laser treatment, hyperthermia, and photodynamic therapy [4,8]. The most common of these is chemotherapy. Our group has recently published a review article that described TNBC and its treatment approaches in more detail [9]. Clinically applicable treatment options for TNBC include anticancer agents, immunotherapeutics, antibody-drug conjugates, and clinically poly-adenosine diphosphate [ADP]-ribose polymerase (PARP) inhibitors (PARPi) [9,10,11]. However, these treatment options are insufficient to efficiently prolong patients’ lives since this disease is heterogeneous and associated with many variables.

Therefore, alternative therapies are required to treat this aggressive disease. Advanced therapies such as combination therapy (immunotherapy with other therapies) and nanotechnology-based therapy could be potentially effective options in the future treatment of TNBC. Combination immunotherapy involves utilizing multiple treatment modalities including immunotherapeutics and chemotherapeutics, immunotherapeutics and DNA damage repair inhibitors, including PARP inhibitors, immunotherapeutics and cell therapeutics, and immunotherapeutics and nucleic acids to treat cancer effectively [12,13]. With the advancements in molecular biology, the combined use of therapeutic agents has become increasingly prevalent. The selection of these therapeutics varies depending on the type of cancer. Specifically, in the context of TNBC, extensive research has focused on combining immunotherapeutics with other agents due to the significant study of programmed death ligand-1 (PD-L1) expression on TNBC cells [14]. Recently, the FDA has granted approval for the use of pembrolizumab (an anti-PD-1 antibody) together with chemotherapy. This approval applies to patients dealing with locally recurrent unresectable or metastatic TNBC, specifically for those whose tumors exhibit PD-L1 expression, as well as for high-risk, early-stage TNBC [14,15]. Numerous preclinical and clinical stage investigations have been conducted, exploring the use of immunotherapeutics together with various other anticancer agents [16].

Over the past few decades, nanotechnology has garnered significant interest among experts in the fields of medical science and engineering due to its minuscule structure and distinctive characteristics, offering potential solutions to certain challenges encountered in medicine [17,18].

Within the realm of drug delivery, varied and diverse types of nanoparticles have been investigated for transporting therapeutics to specific areas in the body where diseased tissues, such as tumors, are located. Moreover, owing to the heterogeneity and distinct characteristics of tumor tissues and their microenvironments—such as leaky vasculature, expression of diverse receptors, and acidity—functionalized nanoparticles hold the potential to effectively target and deliver therapeutics to these locations [19]. Nanoparticles exhibit two distinct modes of targeting cancer: passive targeting is based on the enhanced permeability and retention (EPR) effect; while active targeting utilizes receptors that are overexpressed on cancer cells [20] and other characteristics present inside cancer cells, for example, enzymes and acidic pH.

In this article, we will review recent research on the combination of immunotherapy with other therapies and nanotechnology-based therapies of TNBC in vitro and in vivo as well as clinical trials.

## 2. Combination of Immunotherapy with Other Therapies

**Figure 1:**
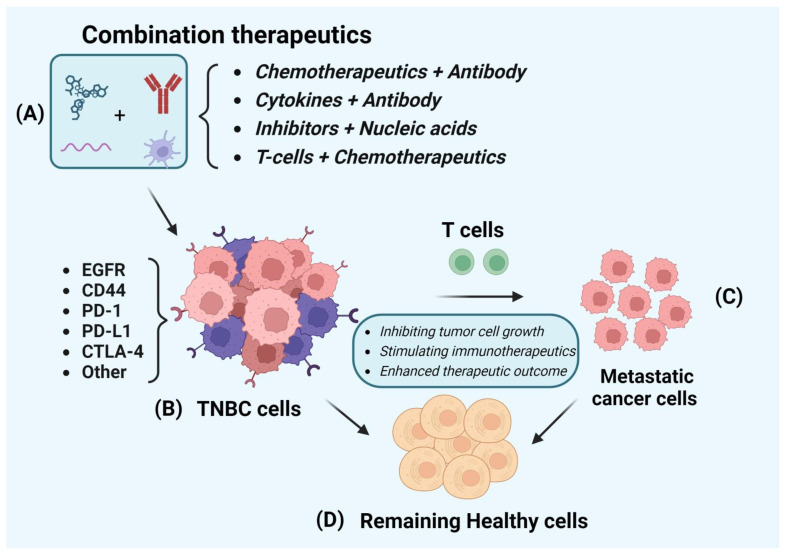
Schematic diagram of combination therapy against TNBC. (**A**) Illustrates a combination of therapeutics; (**B**) presents TNBC cells that express various ligands; (**C**) metastatic cancer cells that are located in other areas than primary tumor tissue; and (**D**) the remaining healthy tissue after combination treatment. Created with Biorender.com, accessed on 6 February 2024.

As previously stated, due to the heterogeneous nature of TNBC and its deficiency in receptors, relying on a single treatment option is nearly impractical. Consequently, numerous studies have concentrated on combining existing therapies with novel approaches to address the challenges presented by TNBC (refer to Table 1). Notably, the most prevalent combinations involve immunotherapeutics paired with other anticancer agents (Figure 1). Figure 1 illustrates the diverse combinations of immunotherapies and other therapies, including the combination of chemotherapeutic agents and antibodies, cytokines and antibodies, inhibitors and nucleic acids, and T cells and chemotherapeutic agents. Combinatorial therapeutics can target TNBC, which possess receptors including EGFR, CD44, PD-1, PD-L1, and CTLA-4, to efficiently destroy it. In addition, the combination therapies have the ability to treat metastatic cancer by producing T cells.

### 2.1. Immunotherapy

Immunotherapy (IMT) represents a category of cancer treatment methods that utilize naturally occurring substances present in the human body or synthesized in the laboratory. These substances are employed to enhance the immune system, enabling the body to identify and destroy cancer cells. IMT has demonstrated encouraging outcomes in the treatment of TNBC [14,37]. The most common forms of immunotherapy are immune checkpoint inhibitors (ICIs), which hinder immunosuppressive receptors such as cytotoxic T lymphocyte antigen-4 (CTLA-4) and programmed death-1 (PD-1). This inhibition enhances the cytotoxicity and proliferative capabilities of tumor-infiltrating lymphocytes (TILs). ICIs, exemplified by monoclonal antibodies, targeting PD-1 (such as pembrolizumab and nivolumab), PD-L1 (like atezolizumab, durvalumab, and avelumab), and CTLA-4 (such as ipilimumab), have demonstrated sustained efficacy across various tumor types [37]. Numerous studies indicate that TNBC exhibits heightened sensitivity to immunotherapy as a result of its distinct characteristics in comparison to other subtypes of breast cancer [4,14]. First, TNBC exhibits an increased presence of TILs, serving as a crucial factor in responding to ICIs and correlating with improved prognosis in early-stage TNBC. Second, elevated PD-L1 expression on both tumor and immune cells in TNBC provides direct targets for ICIs, potentially influencing responses to anti-PD-1 therapies observed in other cancers. Third, TNBC is distinguished by a multitude of nonsynonymous mutations, specifically missense mutations, altering amino acids within proteins. These mutations create tumor-specific neoantigens that can trigger neoantigen-specific T cells, potentially bolstering the antitumor immune response, particularly when augmented by ICIs [37]. Many comprehensive reviews have been reported for immunotherapy in TNBC [14,37,38].

### 2.2. Preclinical Stage Combination Immunotherapy

Cell therapy, especially chimeric antigen receptor (CAR)-T cell therapy, is one potential treatment option for various cancers including TNBC. Researchers have reported several studies using CAR-T cells with other therapeutic approaches against TNBC. For instance, Liu et al. carried out a study using monoclonal antibody (mAb) 2D2 and T cell receptor (TCR)-like (CAR)T cell therapy against TNBC. The mAb 2D2 that was generated was specifically associated with 157–165 New York Esophageal Squamous Cell Carcinoma-1 (NY-ESO-1)157–165 in relation to HLA-A* 02:01, and did not affect any non-A2 or NY-ESO-1-negative cells. Immunogenic cancer antigens such as NY-ESO-1 are one of the most effective for immunotherapy in solid tumors [39,40]. The results showed that 2D2 CAR-T Cells were able to inhibit tumor growth in TNBC tumor-bearing mice [21]. Stuber et al. presented a combination study of receptor-tyrosine-kinase-like orphan receptor 1 (ROR1)-CAR T cells with transforming growth factor β (TGF-β)-receptor signaling inhibitor SD-208 in TNBC. The study results showed that the presence of TGF-β in ROR1-CART cells with TNBC cell culture impaired the cytolytic activity, cytokine production, and proliferation of ROR1-CAR T cells and their viability. The application of the specific kinase inhibitor SD-208 to shield CD8+ and CD4+ ROR1-CAR T cells from the suppressive impact of TGF-β resulted in the consistent maintenance of their antitumor efficacy both in vitro and in the neurophysiologic 3D tumor model [29].

Combinations of radiolabeling or radiotherapy with other therapeutics in TNBC have been reported. McKnight et al. reported a combination therapy of radiolabeled antibody and cytotoxic inhibitor (dasatinib) in TNBC (MDA-MB-468) and patient-derived xenograft (PDX) tumors. Better tumor regression was observed in groups treated with cetuximab (epidermal growth factor receptor-EGFR) and dasatinib (tyrosine kinase inhibitor) in combination compared to groups treated with only dasatinib. The Kirsten rat sarcoma (*KRAS*) mutation may be the reason there was no improvement in MDA-MB-231 xenografts with the addition of cetuximab. The authors concluded that cetuximab-PET can be used to examine the effects of dasatinib on EGFR cellular distribution and the expression of treatment response in wild-type *KRAS* TNBC [24]. Gregor et al. carried out a study using radiation therapy (RT) and ad libitum (AL) diet, and caloric restriction (CR) using in vivo TNBC model. According to the findings, RT resulted in an immune suppressive environment with a significant increase in CD4+CD25+Foxp3+ Tregs, but not in CR mice. Compared to AL+RT mice, the CD8:Treg ratio in CR+RT tumor infiltrating lymphocytes (TILs) was four times higher. According to the findings, combining CR with RT leads to a decrease in intratumoral Tregs, an increase in CD8:Treg ratio, and a rise in PD-1 expression via a process based on CD8 T cells in a TNBC model [26].

Combination studies of chemotherapeutics with immunotherapeutics (ICIs, TKIs, and cellular therapeutics) in TNBC have been widely reported. As an illustration, Singh S et al. demonstrated an immunostimulatory impact by combining low-dose cyclophosphamide with the pharmacological suppression of tumor-associated macrophages (TAMs). They achieved this using either a small molecule inhibitor targeting colony-stimulating factor 1 receptor (CSF1R) or an anti-CSF1R antibody in preclinical syngeneic p53 null mouse models of TNBC. Single-cell RNA sequencing was used to analyze TILs, which include helper T cells and antigen-presenting B cells, that were enhanced in responders to combination therapy. The results revealed that the therapeutic combination was effective in treating several highly aggressive TNBC murine mammary tumor and lung metastasis models [28]. Lee et al. reported a combination therapy of Macitentan and anti-PD-L1 antibody against TNBC, colon, and lung metastatic cancers. The outcomes of the combined therapy demonstrated notable enhancements in antitumor effectiveness, driven by an increase in both the number and activity of CD8+ T cells, alongside a decrease in Treg numbers within tumors and the associated lymph nodes in TNBC, colon, and lung syngeneic tumor models [25]. Wu et al. carried out an in vivo study using anti-PD-L1 antibodies as adjuvant (postoperative) monotherapies for resectable renal cell carcinoma (RCC) and triple-negative breast cancer (TNBC) models in combination with chemotherapeutics as neoadjuvant (preoperative) therapies for resectable TNBC. In the EMT-6/CDDP model, anti-PD-L1 was highly effective for both adjuvant therapy and neoadjuvant therapy when it was combined with paclitaxel chemotherapy (with or without anti-VEGF) [31].

Tentler JJ et al. reported the combination treatment of novel anticancer agent RX-5902 and CTLA-4 or PD-1 inhibitor antibodies in TNBC. The data showed that combining RX-5902 with CTLA-4 or PD-1 antagonists led to a reduction in tumor growth in four T1 and human immune systems and MDA-MB-231 xenograft models. RX-5902 treatment resulted in higher activation of T cells in tumor-infiltrating lymphocytes (TILs) than the vehicle. Also, RX-5902/nivolumab combination significantly enhanced CD4+ T cells in TILs and systemic granzyme B production. The authors found that the effectiveness of nivolumab in a humanized, preclinical TNBC model was enhanced by RX-5902 [30]. Tiara et al. carried out a combination treatment of immunotherapy and chemotherapy (Paclitaxel (PTX)) with positron emission tomography (PET) in TNBC to monitor the therapeutic potential of the combinatorial therapy. The authors used anticancer drug PTX and anti-PD-1 or anti CTL4 antibody as an immunotherapeutic agent against a TNBC model. PET imaging during the combination therapy revealed that PTX improved immune response making it a better environment for IMT [23].

He et al. reported the combination treatment of AEE788 (receptor tyrosine kinases inhibitor) and rapalog (mTOR inhibitors) in TNBC. The findings indicated that the combined therapy effectively suppressed the phosphorylation of mTOR and 4EBP1, mitigated the mTOR inhibition-induced elevation of cyclin D1, and sustained the inhibition of AKT and ERK signaling, consequently enhancing the sensitivity of TNBC cells to rapalogs. The mTOR-interactive *RPS6K* members (*RPS6KA3*, *RPS6KA6*, *RPS6KB1*, and *RPS6KL1*) have been found to be synthetic lethal targets for rapalog combination treatment through the validation of predicted AEE788 targets using siRNA. The authors inferred that their findings offered a prospective strategy involving a combination of multi-kinase inhibitors to counteract resistance to mTOR-targeted therapy in TNBC cells, given the pronounced activation of mTOR signaling in TNBC tumors [41].

Wang et al. performed in vivo CRISPR knockout (KO) screens in syngeneic TNBC mouse models. According to the data, deleting E3 ubiquitin ligase Cop1 in cancer cells leads to a decrease in macrophage-associated chemokines, a reduction in tumor macrophage infiltration, enhanced anti-tumor immunity, and a stronger ICI response. These findings suggest that C/ebpd is the substrate for Cop1 in 4T1 TNBC cancer cells. Cop1 and C/ebpd interact through Trib2, leading to C/ebpd’s ubiquitination and proteasomal degradation [22].

Zanker et al. assessed the local and systemic effects of 3M-052, which is a 7/8 agonist for the intratumoral Toll-like receptor (TLR) in metastatic TNBC models, either alone or in combination with anti-PD-1. The study demonstrated that delivering 3M-052 directly into the tumor reduced its growth, created a T cell-inflammatory tumor microenvironment (TME), and inhibited metastatic spread to the lung. Additionally, 3M-052 was demonstrated to act through dendritic cell activation and the release of type I interferon (IFN) and other pro-inflammatory cytokines, which led to the induction of a T cell-induced TME and the promotion of tumor cell antigen presentation [27].

### 2.3. Clinical Stage Combination Immunotherapy

Several combinatorial studies using various antibodies (ICIs) along with other therapeutics (mostly chemotherapeutics) have been under clinical trials. For instance, Pembrolizumab (anti-PD-1 antibody), Atezolizumab (anti-PD-L1 antibody), Camrelizumab (anti-PD-1 antibody), and Durvalumab (PD-L1 antibody) have been applied in clinical trials as a combination with anticancer agents.

#### 2.3.1. Pembrolizumab (Anti PD-1 Antibody) Combination

Ho et al. reported a phase II clinical trial combination of pembrolizumab (anti-PD-1 antibody) with radiotherapy in seventeen patients with a median age of 52 years old with TNBC. The results showed that the combination therapy had a similar response to pembrolizumab monotherapy in patients with PD-L1-positive mTNBC. In some cases, this combination therapy could be more effective than pembrolizumab alone in the treatment of patients with mTNBC [32]. Schneeweiss et al. performed a phase Ib clinical trial of pembrolizumab with capecitabine (a chemotherapy agent) or PTX (a chemotherapy agent) in 29 patients with TNBC. The trial resulted in longitudinal immunological profiling of the effect of the combination therapy. The patients with mTNBC showed depletion of T cell subsets and reduced T cell clonal richness compared with untreated early-stage breast cancer (ESBC) patient [42]. Anders et al. ran a phase II clinical study in forty patients with pretreated mTNBC using combination of pembrolizumab and cyclophosphamide (Cy, anti-neoplastic agent). The authors first administered Cy intravenously (300 mg/mL) and after one day pembolizumab (200 mg) was given intravenously. The data showed that low-dose Cy was not effective in depleting Tregs prior to anti-PD1 therapy. They found that Clinical Benefit Rate (CBR) was most associated with the expression of B cell signatures in pretreatment tumor specimens. Similar with previous preclinical studies, the results revealed that the B cell response could play an important role in the immune response to ICI therapy in patients with mTNBC [33]. Criscitiello et al. performed a phase I clinical trial using a combination of targeted chemotherapy (PI3K/AKT/mTOR inhibitor) and immunotherapy (PD1/PD-L1 inhibitor) in 151 patients with metastatic breast cancer including 70 of hormone receptor-positive (HR+), 18 of HER2 and 63 of TNBC. The authors found that the patients with TNBC have low outcomes [43].

#### 2.3.2. Atezolizumab (Anti-PD-L1 Antibody) Combination

Iwata et al. carried out phase III IM passion 130 clinical trial in 902 patients with TNBC using a combination of atezolizumab (anti-PD-L1 antibody) with nab-paclitaxel (chemotherapy agent). The trial findings revealed an extended progression-free survival (PFS) in both the intention-to-treat (ITT) population and the subgroup positive for programmed death-ligand 1 (PD-L1), as opposed to placebo plus nab-Paclitaxel. The efficacy of a combination of immunotherapy and chemotherapy agents in Japanese patients with TNBC was consistent with the overall IM passion 130 population [34]. Hecht et al. presented the results of phase Ib study of combination talimogene laherparevec with atezolizumab in 11 patients with mTNBC and 25 patients with metastatic colorectal cancer (mCRC). The trial results verified that the safety profile of intralesional T-VEC and atezolizumab aligns with the anticipated expectations, including the risks associated with intrahepatic injection, albeit with minimal evidence of antitumor activity observed in patients with liver metastases linked to TNBC or CRC [44].

#### 2.3.3. Camrelizumab (Anti-PD-1 Antibody) Combination

Liu et al. conducted an open label phase II clinical trial of combinational treatment of check point blockade (camrelizumab) and antiagiogenesis (apatinib) for 40 patients with TNBC. This combination therapy demonstrated an objective response rate (ORR) significantly superior to those previously reported for anti-PD-1/PD-L1 antibody or apatinib monotherapy. The combination of two agents had positive therapeutic effects and a safe safety profile for patients with advanced TNBC [35]. In another study, Liu et al. carried out a multicenter phase II clinical trial of camrelizumab (anti-PD-1 antibody) combined with Apatinib and Eribulin (antineoplastic) in 46 pretreated patients with advanced TNBC. In patients with advanced TNBC who had been heavily pretreated, the combinatorial therapy trial demonstrated promising efficacy with a measurable safety profile. The ORR of the trial was 37% (17/46, 95% CI 23.2–52.5). The authors mentioned that the favorable ORR and progression-free survival (PFS) were associated with lower tumor PML or *PLOD3* expression in the patients [45]. Zhang et al. performed a phase Ib clinical trial of camrelizumab in combination with apatinib and furuloparib in 32 patients with recurrent or metastatic TNBC. The result revealed no dose-limiting toxicity, 62.1% (95% CI, 42.3–79.3) of the disease control rate (DCR), 52 months (95% CI, 3.6–7.3) of the median PSF, and 64.2% (95% CI, 19–88.8) of 12-month overall survival rate, respectively. Overall results demonstrated that the combination of camrelizumab with apatinib and furuloparib inhibitors showed manageable safety profile in patients with recurrent or metastatic TNBC. Also, the data exhibited low ORR and promising DCR and PFS [46].

Wu et al. performed Future–C Plus phase 2 clinical trial of camrelizumab (anti PD-1 mAb) with famitinib (an angiogenesis inhibitor) and chemotherapy in 48 advanced immunomodulatory TNBC patients. The results showed 81.3% (95% CI, 70.2–92.2) of the ORR and 13.6 months (95% CI, 8.4–18.8) of the median PFS. Also, data revealed that patients with CD8- or PD-L1-positive tumors benefit more from this regimen [47].

#### 2.3.4. Durvalumab (PD-L1 Antibody) Combination

Foldi et al. performed a phase I/II clinical trial of durvalumab (PD-L1 antibody) concomitant with neoadjuvant chemotherapy in 67 patients with an early stage TNBC. The authors used nab-paclitaxel, doxorubicin, and cyclophosphamide as neoadjuvant chemotherapy agents. The results showed that 3-year event-free survival were 78.3% and 71.4% in non-African American (AA) and AA patients and 3-year overall survival was 87% and 81%, respectively. The authors concluded that this combination therapy has similar improved efficacy in early stage TNBC both in AA and non-AA patients [36]. Pusztai et al. carried out I-SPY2 trial (clinical) in 73 patients with stage II/III HER-2-negative BC. According to the I-SPY2 trial’s data, the combination of durvalumab, olaparib, and paclitaxel increased the pCR rate in HER-2-negative BC, which includes TNBC and ER-positive cancers. The combination therapy only worked for a very proliferative and low estrogen receptor, Mamma Print MP2 subset of ER-positive/HER2-negative cancers [48]. Loibl et al. executed a randomized phase II clinical trial of durvalumab (anti PD-L1 antibody) and an anthracycline taxane-based combination in 174 patients with early TNBC. The research results showed that pathological complete response (pCR) rate with durvalumab was 53.4% (95% CI 42.5% to 61.4%) versus placebo 44.2% (95% CI 33.5% to 55.3%). Only patients who received durvalumab alone two weeks before chemotherapy had a significant increase in the pCR rate [49].

#### 2.3.5. Other Immunotherapeutic Combinations

Li et al. studied a phase II, open-label trial of anti-PD-1 antibody (SHR-1210) in combination with apatinib (VEGFR2 inhibitor) for 12 advanced TNBC patients. In preclinical breast cancer models and patients with advanced TNBC, there was evidence that anti-angiogenic therapy and anti-PD-1 antibody have a dose-dependent synergy [50]. Chick et al. a multicenter, randomized, single-blind, phase IIb clinical trial in 587 patients using a combination of the HER2-derived peptide vaccine NPs + trastuzumab (anti HER2 antibody) vs. granulocyte macrophage colony-stimulating factor (GM-CSF) + trastuzumab. The results showed that the combinatorial treatment has a significant benefit in 36-month disease free survival among patients with TNBC, which was not observed in trastuzumab monotherapy [51]. Mohamed et al. studied phase II clinical trial in 54 patients with TNBC using a combination of bevacizumab (anti VEGF-A antibody) with carboplatin and paclitaxel. The trial’s results exhibited 27 months of median PFS (85% confidence interval (CI), 17.019–36.981), and median overall survival (95% CI, 38.973–71.027) [52].

Goal et al. carried out phase III PRESERVE 2 clinical trial of a combination therapy of trilaciclib (intravenous CDK4/6 inhibitor) with gemcitabine (GCb) and carboplatin for 250 patients with mTNBC. A previous phase II study’s data in patients with TNBC demonstrated that administrating trilaciclib prior to GCb resulted in clinically meaningful improvements in overall survival (OS) compared with GCb alone [53]. Wang et al. conducted a phase I clinical trial of DC-CIK immunotherapy with specific chemotherapy (Cy, thiotepa, and carboplatin) in 23 metastatic TNBC patients who had pretreated with anthracyclines and taxanes. According to the authors, the partial response rate was 13%, with stable and progressive disease rates being 56.5 and 30.4%, respectively. They also examined that the average PFS was 13.5 months (95% CI 10.1–16.9 months) and the average OS was 15.2 months (95% CI 12.5–18.1 months). Overall data suggested that the combination therapy was effective and safe for younger mTNBC who were pretreated with anthracyclines and taxanes based adjuvant chemotherapy [54]. Jiang et al. conducted a phase Ib/II FUTURE clinical trial involving seven groups, each combining immunotherapeutic agents and chemotherapy. These groups included pyrotinib with capecitabine, an androgen receptor inhibitor with a CDK4/6 inhibitor, anti-PD-1 with nab-paclitaxel, PARP inhibitor, anti-VEGFR, or mTOR inhibitor with nab-paclitaxel. The trial enrolled 69 patients with refractory metastatic TNBC. The data showed that the ORR was 20 (29.0%, 95% CI 18.7–41.2%) in the patients. Overall, the clinical trial, the FUTURE, revealed that combinatorial therapy of molecular subtyping with targeted sequencing was a promising treatment option for refractory mTNBCs [55].

## 3. Nanotechnology-Based Therapies for TNBC

**Figure 2:**
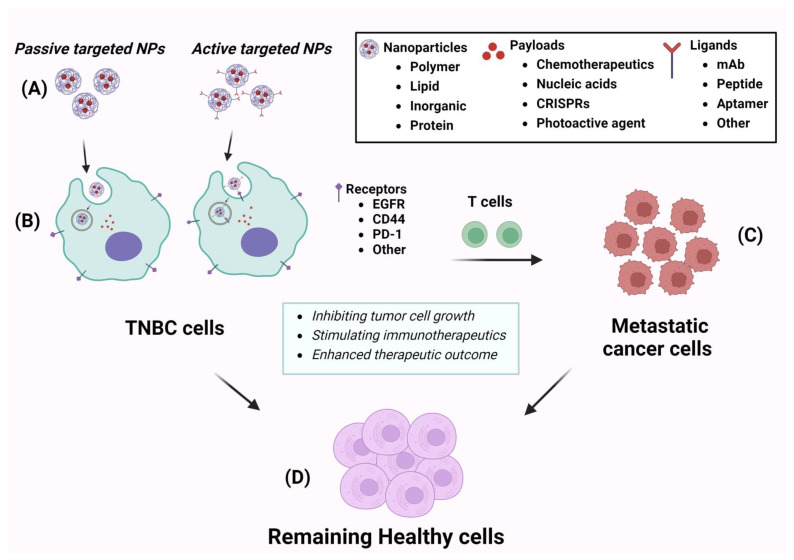
The schematic diagram for nanoparticle-based therapy against TNBC. (**A**) Illustrates drug-loaded nanoparticles; (**B**) presents internalization of nanoparticles through TNBC cells; (**C**) metastatic cancer cells that are located in areas other than primary tumor tissue; and (**D**) the remaining healthy tissue after treatment of nanoparticle-based therapeutics. Created with Biorender.com, accessed on 6 February 2024.

Nanotechnology represents an innovative technology centered around nanoparticles (NPs) characterized by surface charge, small particle size, and structure tailored for precise targeted drug delivery, utilizing a receptor-specific target within cancerous cells [56]. The NPs are crafted from diverse materials like polymers, lipids, proteins, silicon, gold, silver, graphene, and copper [57]. Their fabrication allows for tailoring specific properties based on materials, facilitating ease of targeting sites expressing particular receptors or other stimulants. One of the key advantages of nanoparticles is their ability to carry substantial payloads through encapsulation or incorporation compared to antibody-drug conjugates or polymer-drug conjugates. Table 2 provides an overview of the nanoparticles examined in the treatment of TNBC. Meanwhile, Figure 2 offers a summary visualizing the landscape of nanoparticles in TNBC. As shown in Figure 2, the nanoparticles prepared from various materials (polymer, lipid, inorganic materials, and protein) are capable of carrying various payloads such as chemotherapeutics, nucleic acids, CRISPRs, and photoactive agents. The nanoparticles could be internalized by either of passive or active pathway. In the case of active targeting pathway, the nanoparticles are linked with certain ligands including mAb, peptide, aptamer, and others, allowing the nanoparticles to be taken up by receptor-mediated endocytosis through TNBC cells. On the other hand, plain nanoparticles can be taken up by cells clatrin-mediated endocytosis.

### 3.1. Polymer-Based Nanoparticles

Polymer-derived nanoparticles have garnered extensive exploration as carrier systems in biomedical applications due to their biocompatibility and lack of toxicity [82]. Moreover, polymers are more cost-efficient when compared to lipids and other materials. Numerous polymer-based nanoparticles, derived from either natural or synthetic polymers, have been documented as carriers for delivering anticancer drugs in the treatment of various cancers, including TNBC [82,83].

Krausz et al. developed a doxorubicin (DOX)-containing sol-gel polymer nanoparticle formulation and tested it against DOX-resistant TNBC cell lines. The sol-gel NP formulation is composed of chitosan, polyethylene glycol (PEG), DOX, and tetramethyl orthosilicate HCl. A cytotoxicity study of the DOX-nano hydrogel was performed in DOX-resistant TNBC cell lines, including SUM149 PT, Hs578T, and MDA-MB-157 cell lines. The data showed that the high concentration of DOX-NPs (800 nM) killed more chemoresistant TNBC cells compared with free DOX. However, both groups at a low dose (100 nM) had similar cell-killing activity [58]. Wu et al. developed an indocyanine green (ICG)-loaded glycated chitosan (GC)-based self-assembled nanoparticle (GC@ICG NPs). The authors used GC as a polysaccharide macromolecular immunoadjuvant and ICG as a photoactive agent for combination therapy of phototherapy and immunotherapy. An in vivo study of the GC@ICG NPs showed a strong reduction in tumor growth in the 4T1 tumor-bearing mouse model as well as suppression for lung metastasis, enhancing infiltration of CD8+T cells in distant tumors [84]. Li et al. developed hyaluronic acid (HA)-targeted chitosan nanoparticle loaded with curcumin (CUR). The authors synthesized chitosan (CS)-poly (N-isopropyl acrylamide) that self-assembled to form a nanoparticle due to its amphiphilicity. The CS-NPs were incorporated with CUR and attached HA as a targeting ligand for the CD44 receptor, expressed on the surface of TNBC. The NPs showed better cell-killing effect and high tumor growth suppression activity in the TNBC tumor-bearing mouse model compared with free drug and non-targeted NPs [59].

Sulaiman et al. presented the results of a lipid and poly (lactic-co-glycolic acid) (PLGA) polymer hybrid nanoparticle-based delivery platform co-loading paclitaxel and verteporfin (PV-NP) that was tested against TNBC patient-derived xenograft (PDX) tumor and cancer stem cells (CSCs). The data showed that the dual drug-loaded NPs (lipid-polymer hybrid NPs) significantly inhibited NF-kB, Wnt, and YAP pathways, known to be involved with the growth of TNBC cells, and revealed synergistic effects on killing TNBC bulk tumors and CSCs. The authors performed efficacy studies of NPs in MDA-MB-231 TNBC cells and HCl-002 PDX TNBC tumor model in athymic mice [60]. Chen et al. developed PD-L1-targeted CD155 siRNA (siCD155)-loaded mPEG-PLGA-PLL (PEAL) nanoparticles (NPs) to block PD-L1 and CD155 in a spatiotemporal manner based on their finding of PD-L1 and CD155 receptor co-expression in TNBC. The results showed the combinatorial NPs enhanced early-stage CD8+T cell immune surveillance against 4T1 tumors, but reversed the inhibition status of the late-stage CD8+T cells to prevent 4T1 tumor immune escape [61]. Valcourt et al. carried out research using Notch1-targeted miRNA (miR-34a)-encapsulated PLGA NPs for TNBC treatment. An in vitro study exhibited that the targeted miRNA-polymer NPs had the potential to regulate Notch signaling and downstream miR-34a targets in TNBC, resulting in induced senescence, and reduced cell proliferation and cell migration. The authors used the Notch antibody for two purposes: (i) targeting to Notch receptor expressed on TNBC and (ii) suppressing Notch signaling through signal cascade interference [62]. Valcourt et al. also studied photothermal agent (IR820 dye)-loaded PLGA NPs against TNBC at in vitro and in vivo levels. The results demonstrated that the IR820-PLGA NPs have higher cell-killing activity in the presence of irradiation with 808 nm light against the MDA-MB-231 TNBC cell line, whereas almost no cell killing was observed with treatment without light exposure. An in vivo photothermal study revealed that the IR820-PLGA NPs significantly reduced tumor growth in tumor-bearing mice [63]. Further, Valcourt et al. carried out a study using Notch1 antibody-targeted small molecule drug ABT-737 (Bcl2 inhibitor)-loaded PLGA NPs for TNBC treatment. As shown in the previous report, Notch antibody has dual advantages, including TNBC cell-specific binding and suppression of Notch signaling. The overall results showed that Notch1 antibody-targeted ABT-737-loaded PLGA NPs had a strong targeting ability to TNBC, and efficiently regulated Bcl-2 and Notch signaling to induce cell death in vitro, as well as greatly accumulated in tumor tissue and reduced tumor growth, resulting in high survival of murine [64]. Agnello et al. developed EGFR-targeted cisplatin-loaded PLGA polymer NPs and evaluated against an MDA-MB-231 (TNBC)-bearing tumor nude mouse model. The results indicated that cisplatin-loaded aptamer-targeted NPs held much higher cytotoxicity in tumor cells as compared with free drugs, and significantly reduced tumor growth with higher targeting efficiency and without any signs of systemic toxicity in comparison with free drug and untargeted NPs [72].

Bahman et al. studied dasatinib (tyrosine kinases inhibitor)-loaded poly (styrene-co-maleic acid) (SMA) nanoparticles against both the TNBC cell line and tumor model. Their purpose was to protect dasatinib from fast pharmacokinetic degradation and to prolong its activity by encapsulation in the NPs. The in vitro results exhibited similar cytotoxicity for both NPs and free drug against three cell lines, whereas the tumor suppression efficacy of the NPs in 4T1 tumor-bearing Balb/c mice was 7-fold higher than the same concentration of free drug. The authors claimed that the strong in vivo activity rose by the protection of a SMA micelle system of TKI from the enzymatic degradation [65]. Nabil et al. developed CD44-targeted hydrophobic drug (CFM-4.16)-loaded polymeric NPs to improve drug solubility, tumor accumulation, and anticancer efficacy against TNBC at in vitro and in vivo levels. The authors used hyaluronic acid as a targeting ligand, tocopheryl polyethylene glycol succinate (TPGS) and SMA as the main components of NPs, and momelotinib (MMB; a JAK/STAT inhibitor) as a co-payload. The results showed that CD44-targeted dual drug-loaded polymer NPs selectively delivered the payload to CD44 overexpressing TNBC, resulting in reduced cell viability. An in vivo imaging study revealed higher tumor accumulation of CD44-T-PNPs in TNBC tumor-bearing mice [66]. El-Deeb et al. developed a Taluramycin A-loaded SMA nano micelle (TFA-SMA) with the purpose of reducing the side effects of the drug and increasing tumor accumulation. The results revealed that the nanomedicine holds high anticancer efficacy against TNBC. More importantly, the tumor accumulation of TFA increased four times in the case of TFA-SMA nano micelle in comparison with the free drug in 4T1 tumor-bearing mice [67]. Greish et al. reported cannabinoid (WIN55,212-2) and DOX-co-loaded polymeric NPs (SMA-WIN) and analyzed them in the 4T1 tumor-bearing mouse model. The in vitro cytotoxicity of SMA-WIN and free WIN in three cell lines was similar. However, an in vivo antitumor study against tumor-bearing mice revealed that the combination of DOX and SMA-WIN NPs reduced 60% of tumor growth in comparison with the control group, whereas free DOX and SMA-WIN were able to reduce 34% and 42% tumor growth, respectively [71].

Jiao et al. studied photo-sensitive prodrug polymer nanoparticles (AIP/CPT-NPs) against TNBC. The results showed that combined drug (photosensitizer Al and camptothecin)-loaded NPs induced potent in vivo phototherapeutic damage by 660 nm light exposure, resulting in metastatic tumor suppression in the TNBC model [68]. Xu et al. reported pH-activated nanoparticles encapsulating *POLR2A* siRNA (siPOL2) for the suppression of *POLR2A* expression in TNBC. The data showed that siPOL2 polymer NPs inhibit the expression of the *POLR2A* gene (essential neighboring gene of *TP53*), reducing tumor models as compared to control groups [69]. Zou et al. developed reactive oxygen species (ROS)-responsive galactosylated NPs (DOX@NPs) to inhibit TNBC. The results revealed that DOX@NPs increased tumor cell apoptosis, accumulated efficiently in tumors and suppressed tumor growth in the 4T1 tumor-bearing mouse model by increasing tumor accumulation due to galactose receptor targeting as compared with the control group [70].

Cui et al. developed mitochondria-targeted copper-depleting nanoparticle (CDN) and evaluated them against TNBC. The CDN consists of a copper-depleting moiety (CDM) and a semiconducting polymer NP (SPN). In vitro study of CDN in TNBC cells presented a reduction in oxygen consumption and oxidative phosphorylation, and a decrease in ATP production, resulting in cell apoptosis. An in vivo study in three tumor models showed that CDN inhibited tumor growth, and substantially improved survival, indicating low systemic toxicity [85].

Babu et al. produced hyaluronic acid-coupled cerium oxide-poly(ethyleneimine nanoparticle (CePEI-NPs) with the purpose of using it as a therapeutic agent in TNBC. An in vitro study showed that the HA-CePEI-NPs induced a reduced mitochondrial membrane potential (MMP), which often occurs via the generation of reactive oxygen species (ROS) during the treatment process. They confirmed that the NPs killed the TNBC cells by mitochondria-mediated apoptosis mechanism due to the release of cytochrome c [73].

### 3.2. Lipid-Based Nanoparticles

Lipid-based nanoparticles have been the subject of extensive research as carriers for delivering therapeutic agents. The initial FDA-approved nanoparticle, Doxil, encapsulates Doxorubicin (DOX) within lipid bilayers [19]. A recent milestone in mRNA (messenger RNA) vaccine advancement is closely associated with lipid nanoparticles (LNPs), as they facilitate mRNA delivery to the intended site without degradation [86]. Eskiller et al. developed talazoparib (PARP inhibitor)-loaded solid lipid nanoparticle (SLN) to avoid talazoparib-resistance in TNBC cells. The in vitro study showed that talazoparib-SLN has a strong suppression effect on *MDR1*, *BCRP*, and *MRP1* genes and holds a high protein expression level in comparison with free talazoparib, indicating a promising therapeutic carrier to reverse MDR-mediated resistance in TNBC [74]. Moknlis et al. prepared microRNA (miR-873)-loaded lipid nanoparticle. The authors demonstrated that the miR-873-NPs suppressed *KRAS* mRNA which is a mutated proto-oncogene in TNBC. Also, an in vivo study showed that delivery of miR-873 NPs reduced tumor growth by inhibiting *KRAS* expression in PDAC and TNBC xenograft–tumor models [75]. Zhao et al. developed siRNA and albumin-encapsulated exosome nanoparticles (CBSA/siS100A4@Exosome) with the purpose of treating TNBC. The research data revealed that CBSA/siS100A4@Exosome accumulated in the lung as compared with the same drug-loaded liposome and showed a significant inhibition for the growth of tumor cells owing to the excellent gene-silencing effect [87]. Guo et al. have created a nanolipogel system (tNLG) that is noncationic, deformable, and targeted toward TNBC tumors for CRISPR genome editing. The lipogel tNLGs comprise a shell consisting of two types of noncationic lipids and an alginate core containing 3CR1SPR plasmids. The authors demonstrated that tNLGs are a potent CRISPR knockout of lipocalin 2 (Lcn2), which is a breast cancer oncogene, in human TNBC cells in vitro and in vivo. An in vivo study in an orthotopic TNBC tumor model showed that CRISPR knockout of *Lcn2* reached >81% by tNLGs, resulting in significant tumor growth suppression (>77%) [76].

### 3.3. Inorganic Material-Based Nanoparticle

For the past decade, inorganic nanoparticles have been under scrutiny as drug carrier systems and for their potential as theranostic agents, owing to their minimal toxicity and straightforward preparation [88]. Gold nanoparticles, in particular, have received extensive investigation as both diagnostic agents and carriers for drug delivery [56]. Additionally, iron oxide nanoparticles represent one of the FDA-approved nanoparticle varieties [89].

Ramchandani et al. developed a microRNA (miR-708)-loaded multi-layered gold nanoparticle for the treatment of mTNBC. The data showed that the miR708-NP directly targeted the SOX2/OCT4-mCherry + miR-708^low^ tumor cells to repair metastasis in vivo and had minimal host toxicity, demonstrating great potential for reducing TNBC progression in 4T1 breast tumor-bearing Balb/cJ mice [77]. Oli et al. ran a combinatorial treatment using an immune check point inhibitor with magnetic iron oxide (MIO) nanoparticle hyperthermia against a metastatic TNBC tumor model. Research results showed that although the combination of MIO and immune ICIs (anti-PD-1 and anti CTLA-4 antibodies) reduced tumor volume in 4T1-luciferase cell-implanted *BALB/c* mice as compared to the control group, there was no improvement in the overall survival of mice with the treatment. In addition, the data revealed that a single-fraction application of MION/hyperthermia (HT) combined with immune checkpoint inhibition has the potential to improve metastatic dissemination to the lungs [78]. Zhang et al. developed a hollow mesoporous iron nanoparticle (HFON) encapsulating DOX that can be used as both a therapeutic agent and Magnetic Resonance Imaging (MIR) diagnostic agent for TNBC. An in vitro apoptosis study demonstrated that DOX@HFON induced apoptosis, autophagy, and ferroptosis, resulting in apoptotic cell death in the TNBC cell line. An in vivo antitumor study exhibited that the MAGNET@DOX@HFON group significantly inhibited tumor growth as well as had a great MIR imaging ability in TNBC tumor tissue. The authors concluded that the combinatorial NPs could be potential theranostic agents in TNBC [90].

Fan et al. developed gamabufotalin- and doxorubicin (DOX)-coloaded graphene oxide quantum dots nanoparticles (GTDC NPs). The authors used TAT and RGD peptides as a targeting ligand to tumor tissue. Flow cytometer analysis demonstrated that dual drug-loaded NPs induced more than 89% apoptosis in TNBC cells. An in vivo study showed that the tumor accumulation of targeted dual drug-loaded GTDC NPs increased two times in comparison with naked GTDC NPs, resulting in enhanced antitumor efficacy. Also, the data presented an 84% reduction in lung metastasis through the treatment of NPs against TNBC tumor-bearing mice [79].

Zhang et al. developed a biomimetic nanoparticle from dendritic large pore mesoporous silicon nanoparticles (DLMSNs) and leukocyte/platelet hybrid membrane (LPHM). The NPs were co-loaded with near-infra-red (NIR) fluorescence dye IR780 and a chemotherapeutic agent DOX to use combinatorial therapeutics. An in vitro cytotoxicity study in the presence of laser irradiation exhibited that the LPHM@DDI NPs had a synergistic cell-killing efficacy in TNBC cells, which was raised by inducing apoptosis. The NPs also showed high tumor growth suppression in 4T1 tumor-bearing mice in comparison with control groups [91].

Jiang et al. developed a ZrC nanoparticle in conjunction with radiotherapy (RT) and phototherapy (PT) to enhance antitumor and antimetastatic effects in TNBC. To enhance biocompatibility and targeting ability, the authors coated the nanoparticles with bovine serum albumin (BSA) and attached folic acid to the NPs. The size of NPs was approximately 100 nm. An in vitro immunofluorescence study revealed more DNA damage and more ROS generation in TNBC cells through ZrC NP treatment. Further, in vitro and in vivo studies showed that ZrC NPs with a combination of RT and PT had a strong cell-killing effect and tumor-suppressing activity in the TNBC tumor-bearing mouse model [92].

### 3.4. Peptide and Protein-Based Nanoparticle

Nanoparticles derived from peptides and proteins have been studied as drug delivery systems of varied and diverse therapeutics due to their biocompatibility and minimal toxicity. An instance of this is Abraxane, an FDA-approved nano-complex composed of albumin and Paclitaxel, applied for the treatment of several cancer types [93].

Egorova et al. created NPs that are based on peptides and targeted to chemokine receptor-4 to deliver siRNAs that suppress major transduction pathways in combination. Combinatorial transfection of anti-*COL4A2* and anti-CDC20 siRNAs NPs resulted in a 1.5–2-fold inhibition of TNBC cells’ proliferation and migration. The authors concluded that CXCR4 ligand-modified L1-polyplexes containing *AQP3*, *CDC20*, and *COL4A2* siRNAs have a huge combinatorial effect on inhibiting the proliferation of TNBC cells [94].

Yuan et al. carried out a study using Abraxane (paclitaxel-loaded albumin NPs) along with Taxol as a control against TNBC. The in vitro study results showed that both Abraxane and free Taxol had similar cytotoxicity in TNBC cell lines. In the case of the in vivo study, although Abraxane held 3–5-fold lower blood drug concentration in comparison with Taxol; they exhibited similar tumor suppression effects in the orthotopic breast cancer *NOD/SCID* mouse model. In contrast, Abraxane reduced breast cancer stem-like cells (CSCs) frequency by 3 to 9-fold, while Taxol enhanced breast CSCs frequency in the tumor model. In addition, the data showed that Abraxane improved intracellular uptake in cancer cells as compared to Taxol by 3 to 15 times, indicating Abraxane’s superior efficacy against CSCs to Taxol [95].

Wang et al. developed a peptide-drug conjugate (PTX-SM-TAR) comprising PTX and a fused peptide TAR containing a tumor-targeting peptide, A7R, and a cell-penetrating peptide, TAT. The PTX-MS-TAR conjugate self-assembled into nanoparticles. The in vitro study of PTX-SM-TAR NPs exhibited higher binding affinity to NRP1 (Neuropilin 1, tumor angiogenesis receptor), great transvascular transport, and tumor penetration ability. An in vivo antitumor efficacy study showed that PTX-SM-TAR NPs had a tumor inhibition rate of 43.24%, whereas PTX and TAR groups reveal 28.47% and 7.81% tumor inhibition rates, respectively, against a 4T1 cell-based TNBC tumor model [80].

Liu et al. described cyclic arginyl-glycyl-aspartic acid (cRGD) peptide-targeted human serum albumin (HSA) nanosystem with the purpose to co-deliver albendazole (ABZ) and iodine-131 (^131^I) for chemoradiotherapy of TNBC. The NPs were prepared by self-assembling HSA in the presence of ABZ and by attaching cRGD and ^131^I to the surface of the NPs. An in vitro cytotoxicity study exhibited that the NPs had high cell-killing activity and reduced cell migration in MDA-MB-231 and 4T1 cells compared with free drug and non-targeted NPs. In vivo, the targeted and radiolabeled NPs suppressed tumor growth in 4T1 tumor-bearing mice and were able to accumulate in tumor tissue [81].

## 4. Conclusions

Numerous studies conducted over the past few decades have explored various treatment approaches for Triple-Negative Breast Cancer (TNBC), including chemotherapy, immunotherapy, phototherapy, radiotherapy, and targeted therapy. However, the heterogeneous nature and limited receptor expression of TNBC have rendered monotherapies ineffective. Recent successful clinical trials have focused on combination therapies, such as the combination of immunotherapy (ICIs) with chemotherapy or targeted therapy. Some of these combination therapies have received approval from the US FDA. This review article provides a comprehensive summary of recent efforts in research both in preclinical and clinical stages of combination therapies, encompassing immunotherapy with chemotherapy, immunotherapy with other therapies, chemotherapy with targeted therapy, as well as preclinical nanotechnology-based therapy for TNBC.

Combination therapy encompasses the pairing of immune checkpoint inhibitors (ICIs) with chemotherapeutics, cyclin-dependent kinase inhibitors (CKIs) with chemotherapeutics, targeted therapeutics with chemotherapeutics, and radiotherapeutics with chemotherapeutics. Nanotechnology-based therapy involves the utilization of diverse materials of formulation, including those made of polymers, lipids, inorganic materials, and proteins. These nanoparticles have the capability to transport a range of therapeutic agents, such as anticancer drugs, nucleic acids, small molecule inhibitors, and other biologics. Moving forward, a substantial percentage of cancer treatment options, including TNBC, are anticipated to involve combination therapies. Furthermore, nanotechnology-based therapies are poised to progress into clinical trials and gain approval for applications.

While combination immunotherapies with other therapies have demonstrated promising outcomes, further research is required to explore additional combinations that can extend patients’ lives. The integration of AI and drug delivery systems holds promise for developing more potent therapeutic combinations in the future. Specifically, in the context of nanoparticle-based therapies for cancer treatment, increasing the number of clinical stage studies is imperative to enhance the likelihood of reaching the approval stage. This necessitates conducting optimization studies in both academic and industrial settings to refine these therapies and maximize their efficacy.

## Figures and Tables

**Table 1:** Summary of combination between immunotherapy and other therapies.

Comb Therapeutics	Cell Line and Model	Stage	Results	Molecular Target	Ref.
Monoclonal antibody 2D2 and TCR-like CAR-T cell	HEK293T, MDA-MB231-ESO1, PC3-A2-ESO1T2, Mel586, Mel624, Mel1558 cells; MDA-MD231-N4-ESO-1	In vitro and in vivo	TCR-like antibody derived CAR-T cells were able to inhibit tumor cell growth and overall survival of mouse.	HLA-A2, NY-ESO-1	[21]
Anti-PD1 antibody and CRISPR knockout	4T1-tumor bearing mice model	In vitro and in vivo	In vivo CRISPR knockout enhanced antitumor immunity and strengthened immune checkpoint blockade	E3 ubiquitin ligase Cop1	[22]
ICIs and PTX	4T1, EO771; 4T1 and E0771 tumor-bearing mice model	In vitro and in vivo	The combination treatment reduced tumor growth.	PD-1 and CTLA-4	[23]
Dasatinib/radiotracer-attached cetuximab	MDA-MB231 cell and MDA-MB468 PDX tumor model	In vitro and in vivo	The results showed that combination of radiolabeled antibody and dasatinib was able to monitor drug distribution and treatment response in KRAS TNBC.	EGFR	[24]
Macitentan/anti-PD-1 antibody	MDA-MB231, 4T1, CT26 and LL/2, EMT6; MDA-MB231, 4T1, and EMT6 tumor-bearing mice	In vitro and in vivo	The combination of MAC and anti-PD-1 antibody showed strong antitumor effect against TNBC, colon and lung cancer.	PD-1, CD8+T endothelin receptor	[25]
Radiotherapy/caloric restriction ad libitum diet	4T1-tumor bearing mice model	In vitro and in vivo	The results revealed that the combination RT and CR enhanced immunotherapy effect against TNBC.	CD+8T cell, TME	[26]
3M-O52 and anti PD1 antibody	E0771, CAL-120 and MDA-MB-231cells; 4T1.2 or E0771 bearing mice	In vitro and in vivo	The results showed the combination treatment reduced tumor growth and metastatic spread to lung	IFN, TME, PD1, Toll-like receptor 7/8	[27]
Cyclophosphamide (Cytoxan) and CSF1R inhibitor or an anti-CSF1R antibody	T11, T12 cell lines; T11, T12 and 215/R tumor-bearing mice model	In vitro and in vivo	The results illustrated the complexity of the tumor immune microenvironment and highlight different immune responses that result from rational immunotherapy combinations.	CSF1R	[28]
CAR-T cell and TGF-B inhibitor SA-208	MDA-MB-231	In vitro and in vivo	The results showed the combinatorial treatment of CAR-T cell and TGF-B receptor blockade was able to suppress tumor growth.	ROR1, TGF-B receptor	[29]
RX-5902 and PD-1 or CTLA-4 combination	4T1 and MDA-MB231 TNBC tumor bearing mice model	In vitro and in vivo	The combination treatment decreased tumor growth and increased activated T cells	CTLA-4/PD-1	[30]
Anti-PD-L1 antibody and sunitinib/Paclitaxel	EMT-6/P, EMT-6/CDDP, REN CA,	In vitro and in vivo	In the EMT-6/CDDP model, combination of anti-PD-L1 with paclitaxel chemotherapy (with or without anti-VEGF) was most effective as a neoadjuvant therapy in breast cancer.	PD-L1/VEGF/VEGFR2	[31]
Pembrolizumab and radiotherapy	17 patients with TNBC	Phase II trial	Neutral efficacy but encocering? clinical activity	PD-L1	[32]
Pembrolizumab and cyclophosphamide (antineoplastic agent)	40 patients with TNBC	Phase II	Low outcome for TNBC patients		[33]
Aterolizumab and nabpaclitaxel	902 patients with TNBC	Phase III	Consistent with the overall IM passion 130 population	PD-L1	[34]
Camrelizumab and Apatinib	40 patients	Phase II	Objective response rate was much higher than monotherapy	PD-L1	[35]
Durvalumab and nab-paclitaxel, DOX, and Cyclophosphamide	67 patients with early stage TNBC	Phase I/II	The combination treatment improved survival rate of the patients.	PD-L1	[36]

**Table 2:** Summary of nanotechnology-based therapy (therapeutic agent-loaded nanoparticles and their efficacy against TNBC).

Nanoparticle	Drug	Cell Line and Model	Stage	Results	Targeting Moiety and Receptor	Ref
Sol-gel polymer nanoparticle	DOX	SUM149PT, HS578T, MDA-MB157	In vitro and in vivo	DOX-NPs showed higher cell killing activity in comparison to free DOX.	EPR	[58]
HA-coated chitosan NPs	Curcumin	4T1 cell line, 4T1 tumor-bearing mice	In vitro and in vivo	It exhibited higher antitumor efficacy in TNBC-tumor model.	CD44 receptor	[59]
Lipid-polymer hybrid nanoparticle	PTX and verterporfin	MDA-MB231 cell line, HCl-002 PDX TNBC mice model	In vitro and in vivo	As compared free drugs, the NPs showed significant suppression for tumor growth.	NFkB, Wnt and VAP pathways, cancer stem cells	[60]
mPEG-PLGA-PLL NPs	siRNA CD155	4T1 cell line, 4T1 –orthotopic tumor model	In vitro and in vivo	The NPs improved early stage CD8+T cell immunosurveillance.	PD-L1 and CD155 receptor	[61]
PLGA Nps	miRNA	MDA-MB231 TNBC cell, MCF-10A normal cells	In vitro and in vivo	The NPs was able to impair TNBC cells	Notch-1 signal, miR-34a downstream	[62]
PLGA NPs	IR820 dye	MDA-MB231 cell, Tumor-bearing mice	In vitro and in vivo	The NPs significantly reduced TNBC tumor growth.	EPR	[63]
PLGA NPs	ABT-737 (BcL2 inhibitor)	MDA-MB231 cell line, MCF-10A normal cell	In vitro and in vivo	The NPs exhibited high tumor accumulation and strong inhibition of tumor growth in TNBC tumor model.	Notch—1 signal targeting	[64]
SMA polymer NPs	Dasatinib (TKI)	MDA-MB231, MCF7, and 4T1 cells; 4T1-bearing tumor model	In vitro and in vivo	The NPs showed 7-fold higher tumor suppression effect than free drug in tumor-bearing mice model.	EPR, ABL kinase Src (Kin2) receptor TKI	[65]
TPGS-SMA polymer NPs	CFM-4.16/momelotinib	MDA-MB-231, MDA-MB468; MDA-MB231-bearing mice model	In vitro and in vivo	The NP revealed strong targeting ability to CD44 expressing cell	CD44 receptor	[66]
SMA polymer micelle	Taluzamycin-A	MDA-MB-231, MDA-MB468, MCF-7 cells; 4T1-tumor bearing mice	In vitro and in vivo	The NPs were taken up by tumor tissue 4-times greater than free drug.	EPR	[67]
Polymer NPs	AL/camptothecin	4T1-cell line 4T1-tumor bearing mice model	In vitro and in vivo	The combinatorial drug-loaded NPs showed tumor suppression effect against metastatic TNBC.	EPR, light sensitive delivery	[68]
PLGA polymer and lipid hybrid NPs	siRNA	MDA-MB453, MDA-MB231 cells; MDA-MB453 and MDA-MB231 –bearing mice model	In vitro and in vivo	The results showed that the NPs inhibited *POLR2A* and significantly reduced POLR2A-positive tumor growth	*POLR2A*	[69]
P-glactose—polymethacrylate NPs	DOX	Human MDA-MB231, 4T1, HUVEC cells; 4T1 tumor-bearing mice	In vitro and in vivo	DOX-loaded NPs revealed higher cellular uptake and tumor accumulation as well as tumor suppression effect	Glactose, EPR	[70]
SMA-WIN polymer NPs	DOX and canabinoid	MDA-MB231, 4T1, MCF7; 4T1-bearing mice	In vitro and in vivo	The dual drug-loaded NPs significantly reduced tumor growth as comparison in free drugs.	EPR	[71]
PLGA NPs	Cisplatin	MDA-MB231, BT-549, and MDA-231-EGFR-KO cells; MDA-M231 and MDA-MB231-KO-bearing mice	In vitro and in vivo	The cisplatin-PLGA Nps revealed strong tumor suppression efficacy in TNBC mice model	EGFR	[72]
HA-CePEI NPs	Cerium oxide (Ceria)	MDA-MB231, and HBL-100 cells	In vitro and in vivo	The NPs showed a strong apoptotic effect in TNBC cells due to its ROS generation and targeting ability	CD44	[73]
SLNPs	PARP inhibitor talaroparib	HCC1937, MCF10A, and HCC1937-RC cells	In vitro and in vivo	The NPs were able to reduce *MDR1*, *BCRP*, and *MRP1* gene expression, leading efficient therapeutic activity.	EPR	[74]
LNPs	microRNA (miR-878)	MCF10A, MDA-MB436, MDA-MB231, MDA-MB453, BT-20, HCC1937, SKBR3, T47D, HEK293 noraml HPDA, PANC1, BxPC3, MiaPaCa-2, Capan-2	In vitro and in vivo	The NPs inhibited tumor growth in PDAC and TNBC tu tumors by suppressing cell proliferation and inducing apoptosis	EPR	[75]
Lipogel tNLGs	3CRISPR plasmid	MDA-MB231, MDA-MB436, MCF10A; MDA-MB231-tumor-beairng mice	In vitro and in vivo	The NPs suppressed the expression of LCN2 oncogene and inhibited minimal host toxicity	ICAM1	[76]
LbL-coated Gold NPs	miRNA (miR-708)	MDA-MB231, 293T, MDA-MB231-LM2 cells; 4T1-tumor-bearing mice	In vitro and in vivo	miRNA-gold NPs exhibited minimal host toxicity	EPR	[77]
Magnetic iron oxide NPs	Immune check point inhibitor	4T1 cell line and 4T1-tumor bearing mice	In vitro and in vivo	The MIO NPs reduced tumor growth in TNBC tumor model	EPR, PD-1, CTLA-4	[78]
Graphene oxide Qdot NPs	Gamma bufotacin and DOX	MDA-MB231, BGC-823, Hela, NIH-3T3 RAW264.7	In vitro and in vivo	The dual drug-loaded NPs were taken up 2-fold higher by tumor cells in comparison with naked one and reduced lung metastasis.	TAT, RGD	[79]
Peptide-drug conjugate NPs	PTX	4T1-mcherry-luc cell; 4T1-tumor bearing mice	In vitro and in vivo	The NPs strongly inhibited tumor growth	NRP1 (Neuropilin 1)	[80]
RGD-HAS NPs	Aldendarole/iodine-131	MDA-MB231; 4T1-cells	In vitro and in vivo	RGA-coupled NPs were able to penetrate into tumor and inhibit tumor growth	cRGD and integrin	[81]

## Data Availability

The data presented in this study are available in this article.
